# A description of self-medication with cannabis among adults with legal access to cannabis in Quebec, Canada

**DOI:** 10.1186/s42238-022-00135-y

**Published:** 2022-05-26

**Authors:** Antoine Asselin, Olivier Beauparlant Lamarre, Richard Chamberland, Sarah-Jeanne McNeil, Eric Demers, Arsène Zongo

**Affiliations:** 1 Faculty of Pharmacy, Unversité Laval, Quebec City, QC Canada; 2 Population Health and Optimal Health Practices Research Unit, CHU de Québec - Université Laval Research Centre, 1050 Chemin Ste-Foy (J1-14B), QC G1S 4L8 Quebec City, Canada

**Keywords:** Medical cannabis, Recreational cannabis, Self-medication, Online survey, Quebec

## Abstract

**Objective:**

Cannabis is increasingly used for medical purposes, particularly in countries like Canada where cannabis was recently legalized for recreational use. We aimed to assess self-medication with cannabis post-cannabis legalization among adults in the Canadian province of Quebec.

**Methods:**

This is a cross-sectional online survey of a self-selected convenience sample conducted in Quebec, Canada, from November 2020 to January 2021. Individuals aged ≥ 21 years who endorsed using cannabis bought in legal recreational cannabis stores to self-medicate a health condition were included. Data were analyzed using descriptive statistics and stratified according to sex, age, and the type of cannabis use (exclusively medical versus medical and recreational use).

**Results:**

Four hundred eighty-nine participants were included. The median age was 34 years, and 48% were women. About 25% reported exclusive medical use of cannabis. Treated conditions included anxiety (70%), insomnia (56%), pain (53%), depression (37%), and many others. Reasons for not consulting in cannabis clinics included lack of information (52%), the complexity of the process (39%), accessibility of cannabis clinics (23%), and others.

Tetrahydrocannabinol (THC) dosage > 20% was reported by 32%. Smoking was the main route of use (81%). Possession of prescribed drugs was reported by 56%. Professionals consulted for information on cannabis included recreational cannabis store agents (36%), physicians (29%), and others.

Overall, significant differences were observed for many of the comparisons according to sex, age, and the type of cannabis use.

**Conclusions:**

Many conditions are self-medicated with cannabis. The use of high doses of cannabis, smoking as a preferred method of use, and concurrent use of other medications may pose some risks to individuals. Addressing the reported barriers to medical access to cannabis is urgently needed.

**Supplementary Information:**

The online version contains supplementary material available at 10.1186/s42238-022-00135-y.

## Contributions to knowledge

What does this study add to existing knowledge?

- This study provides a detailed description of self-medication with cannabis, an important public health issue.

What are the key implications for public health interventions, practice, or policy?

- Public health and policy implication: The results suggest an urgent need for interventions and actions to address barriers to cannabis access through the healthcare systems
- Practice: Results are relevant to healthcare professionals to assess the possible use of cannabis among their patients with similar characteristics and conditions and to prevent possible cannabis-drug interactions and other cannabis-related risks.

## Introduction

The use of cannabis to manage health conditions or symptoms is increasing worldwide. Particularly, the legalization of cannabis for both medical and recreational use in some US states and in Canada has facilitated self-medication with cannabis (Sarvet et al. 2018; Statistics Canada 2020). In Canada, cannabis was legalized for medical use in 2001with some restrictive conditions (Health Canada 2020). In October 2018, Canada legalized the production, sale, and consumption of cannabis for recreational purposes (Ministry of Justice (Canada) n.d.). The province of Quebec (the government) thus decided to have a legislated monopoly on the sale of recreational cannabis within the province through a public not-for-profit company called the *Société Québécoise de Distribution du Cannabis* (SQDC) (Gouvernement du Quebec n.d.; Société Québécoise Du Cannabis (SQDC) n.d.). The province also raised the legal age of cannabis access to 21 years as opposed to 18 years in the rest of the country (Gouvernement du Quebec n.d.; Société Québécoise Du Cannabis (SQDC) n.d.). No further restriction for access via the SQDC was implemented. Cannabis can be purchased in SQDC’s stores in person or via the Internet. A post-legalization cannabis survey in the province of Quebec in 2019 showed that cannabis was used to treat a health condition by 23.5% of cannabis users (Roy and Conus 2020). However, only 4.6% of participants reported acquiring their cannabis from producers licensed by Health Canada, suggesting that the majority of those who reported medical use were acquiring their cannabis from the recreational market, relatives, or illegal sellers. A 2019 Canadian survey of medical cannabis users also showed that 45% of participants never had a medical document for their cannabis (Canadian Pharmacists Association (CPhA) n.d.). The main sources of the acquisition were licensed producers (38%), informal (37%), and legal recreational cannabis stores (47 %) (Canadian Pharmacists Association (CPhA) n.d.). A study carried out by Sexton et al. in the USA found that 60% of respondents who reported a medical use of cannabis used cannabis without medical advice (Sexton et al. 2016).

Self-medication with cannabis could present some risks for users. Main concerns include possible intoxication for non-standardized or high cannabis dosage (Volkow et al. 2014) and users experiencing known and unknown adverse events (Volkow et al. 2014) including cannabis-drug interaction for those with prescribed medications (Vazquez et al. 2020). This is particularly critical considering the lack of minimal benefit-risk assessment in the context of self-medication. For all these reasons, a description of the phenomenon that would help to understand the problem and implement solutions is needed. However, although surveys have documented cannabis use by distinct patient groups, very few have specifically targeted individuals using cannabis for self-medication, particularly post-recreational cannabis legalization in Canada. Considering the increasing popularity of the therapeutic use of cannabis and the potential risks associated with its use, the present study was designed to examine the global portrait of self-medication with cannabis in the province of Quebec including users’ characteristics, reasons for self-medication, conditions treated, patterns of cannabis and other drug use, and healthcare and other resources utilization.

## Methods

### Study design

This is a cross-sectional study conducted through an online survey that took place between November 10, 2020, and January 31, 2021, in Quebec, Canada.

### Study population

All individuals aged 21 years or older (i.e., legal age to access cannabis in the province of Quebec) were eligible to complete the survey if they spoke French and reported self-medication with cannabis acquired at the *Société québécoise du cannabis* (SQDC). SQDC is a public not-for-profit society that has a monopoly for recreational cannabis sales within the province of Quebec since the legalization of recreational cannabis in 2018 (Gouvernement du Quebec n.d.; Société Québécoise Du Cannabis (SQDC) n.d.). We defined self-medication with cannabis as the use of cannabis-based products for the prevention, treatment, or alleviation of symptoms of a physical or psychological illness.

### Data collection

Data were collected through LimeSurvey, a web-based platform. The study questionnaire was developed by the research team and comprised 48 questions grouped in six sections. The questions were developed after a careful review of prior cannabis-related surveys, studies on cannabis efficacy and safety, etc. This step allowed to build a pool of questions that were reviewed by the research team to obtain a version that was next pre-tested. The questionnaire pre-test was done with a small number of participants. Following the pre-test, the questionnaire was revised to have a final version. Revision included rewording some questions, changing some questions’ order in the questionnaire, etc. (the French version of the questionnaire is provided in supplemental materials). Section 1 of the questionnaire was about the source of cannabis supply and the reasons for consumption (recreational or medical), the objective being to exclude participants who used exclusively cannabis for recreational purposes. Section 2 was composed of questions about demographic characteristics (age, gender, income, administrative region of the participant, etc.). Section 3 focused on conditions treated and pattern of cannabis use (conditions treated, variety of cannabis, tetrahydrocannabinol (THC) and cannabidiol (CBD) dosages, frequency of consumption, route of administration, and use of other drugs). Anticipating that a majority of participants could use cannabis for more than one condition, we decided not to ask to report cannabis use patterns for every single condition to reduce the burden of completing the questionnaire and most importantly to minimize recall bias. Section 4 of the questionnaire assessed the perceived effects of cannabis (not reported in this manuscript). Section 5 inquired about barriers to accessing the medical cannabis circuit. The last section assessed the perceived role of healthcare professionals.

Three questions were added after the launch of the survey and were thus completed by a subset of the participants. These questions assessed (1) the declaration of cannabis use to healthcare professionals, (2) the need to have access to healthcare professionals for advice related to their medical use of cannabis, and (3) the impact of the COVID-19 pandemic on cannabis consumption for participants who were using cannabis before the pandemic.

The recruitment was promoted through social media (mainly Facebook), patients’ associations such as Quebec Association for Chronic Pain, and leaflets distributed in pharmacies. An online consent form had to be read and agreed upon by all participants before accessing the study questionnaire. The study inclusion criteria were assessed in the first section of the questionnaire. Participants who declared at least one exclusion criterion were automatically excluded from the survey and their questionnaires were marked as completed.

### Statistical analysis

The analyses were mainly descriptive and consisted of calculating proportions for categorical variables and mean and median for continuous variables. First, we analyzed the characteristics of the participants that we stratified by gender and the type of cannabis use (exclusively medical or mixed medical and recreational use). Next, we described the conditions treated with cannabis, the reasons for self-medication, the patterns of cannabis use, and other medications used concomitantly. To further describe the portrait of cannabis use for self-medication, we also stratified the analysis by some characteristics such as age and gender to highlight differences or similarities that may exist between certain groups. For stratification by gender (women, men, and other gender), we could only consider men and women categories as a small number of participants was observed in the other gender category. Statistical comparisons were done using the chi-square test. *p*-values below 0.05 were considered statistically significant. Pairwise comparisons were conducted for categorical variables (> 2 categories) with a significant global *p*-value using a Bonferroni correction for multiple comparisons. The data were analyzed with SAS version 9.4 (SAS Institute, Cary, NC, USA).

## Results

### Participant’s characteristics

Of 660 participants who consented to participate in the study, 489 satisfied the inclusion criteria and were included for analysis. Of the 171 individuals who were excluded, one was under 21 years old, 98 reported recreational cannabis use only, and 72 did not use cannabis from SQDC (most likely from illegal sources or medical cannabis circuit). Most of the participants (81%) reported having reached the survey from social media, 14.5% from the Université Laval mailing list, and the remaining from other sources. The mean age of the 489 participants was 36 years (age range: 21–77 years), 48.67% were women, 48.06% were men, and 3.27% reported other gender (Table 1). Participants were distributed in all the regions of Quebec. The quasi-totality of respondents was Caucasians (93.57%). About 25% (*n* = 122) reported using cannabis only for medical purposes while the other 75% declared using cannabis for both recreational and medical purposes. Almost half of the respondents who use cannabis for both purposes were under 30 years (47.54%) while this proportion was 26.23% among the exclusive medical users. Women reported exclusive medical use more frequently compared to men (Bonferroni *p*-value: 0.01625). Participants aged 21–30 years were more likely to report mixed cannabis use compared to individuals aged 51–60 years (Bonferroni *p*-value = 0.00006) and those >60 years (Bonferroni *p*-value = 0.00023) (Table 1 and Supplemental Table 1).

**Table 1:** Characteristics of participants who reported self-medication with cannabis based on an online survey in Quebec from November 2020 to January 2021 (*n* = 489)

Characteristics^**a**^	Gender^**b**^		***P***-value*	Type of cannabis use		***P***-value*	Total participants (***n*** = 489) ***N*** (%)
	Men (***n*** = 235)	Women (***n*** = 238)		Medical use only (***n*** = 122)	Medical + recreational use (***n*** = 367)		
**Gender**						**0.0173**	
Male	-	-		46 (37.70)	189 (51.50)		235 (48.06)
Female	-	-		73 (59.84)	165 (44.96)		238 (48.67)
Others	-	-		3 (2.46)	13 (3.54)		16 (3.27)
**Age (mean = 36 years, min = 21, max = 77, median = 34)**			0.1419			**<.0001**	
21–30	93 (39.74)	100 (42.02)		32 (26.23)	174 (47.54)		206 (42.21)
31–40	69 (29.49)	56 (23.53)		33 (27.05)	95 (25.96)		128 (26.23)
41–50	45 (19.23)	37 (15.55)		23 (18.85)	59 (16.12)		82 (16.80)
51–60	18 (7.69)	31 (13.03)		22 (18.03)	27 (7.38)		49 (10.04)
> 60	9 (3.85)	14 (5.88)		12 (9.84)	11 (3.01)		23 (4.71)
**Ethnicity**			**0.0206**			0.7377	
Caucasian	224 (96.55)	214 (91.45)		114 (94.21)	337 (93.35)		451 (93.57)
Others	8 (3.45)	20 (8.55)		7 (5.79)	24 (6.65)		31 (6.43)
**Marital status**			0.7213			0.7222	
Single	106 (46.29)	104 (44.64)		53 (44.92)	168 (46.80)		221 (46.33)
In a relationship (married or unmarried)	123 (53.71)	129 (55.36)		65 (55.08)	191 (53.20)		256 (53.67)
**Highest level of education**			**0.0038**			0.7900	
Primary or high school	53 (23.25)	40 (17.02)		23 (19.49)	72 (19.95)		95 (19.83)
Technical school	62 (27.19)	41 (17.45)		27 (22.88)	78 (21.61)		105 (21.92)
College	68 (29.82)	77 (32.77)		35 (29.66)	116 (32.13)		151 (31.52)
First cycle university	31 (13.60)	58 (24.68)		22 (18.64)	73 (20.22)		95 (19.83)
Second or third cycle university	14 (6.14)	19 (8.09)		11 (9.32)	22 (6.09)		33 (6.89)
**Annual income**			0.1046			0.4683	
< 10,000	9 (4.00)	16 (7.14)		6 (5.31)	20 (5.68)		26 (5.59)
10,000–24,999	36 (16.00)	53 (23.66)		29 (25.66)	70 (19.89)		99 (21.29)
25,000–49,999	74 (32.89)	58 (25.89)		25 (22.12)	111 (31.53)		136 (29.25)
50,000–74,999	38 (16.89)	42 (18.75)		23 (20.35)	58 (16.48)		81 (17.42)
75,000–99,999	35 (15.56)	24 (10.71)		13 (11.50)	46 (13.07)		59 (12.69)
100,000–124,999	17 (7.56)	12 (5.36)		9 (7.96)	20 (5.68)		29 (6.24)
> 125,000	16 (7.11)	19 (8.48)		8 (7.07)	27 (7.76)		35 (7.53)

*See supplemental Table 1 for pairwise comparisons for categorical variables with a significant global *p*-value

^a^Missing answers are excluded from percentages

^b^The category “other gender” was not considered for the stratification by gender due to the low number of participants in this category

### Reasons for self-medication with cannabis

The main reasons reported for not consulting cannabis clinics were the lack of information on the medical access of cannabis (52.85%), the perceived complexity of the process (39.86%), the difficulty accessing a cannabis clinic (23.23%), the inconvenience of the follow-up with a physician for cannabis (20.27%), the inability to choose the cannabis products (18.91%), the delay to obtain cannabis in the medical circuit (16.17%), and the price (12.07%) (Table 2). In general, men and participants under 34 years were more likely to report a reason limiting their access to medical cannabis (Table 2).

**Table 2:** Reasons for not seeking care in cannabis clinics among individuals who reported self-medication with cannabis in an online survey in Quebec from November 2020 to January 2021 (*n* = 489)

Reason	Gender^**ab**^			Age^**b**^			Type of cannabis use			Total
	Men (***n*** = 210)	Women (***n*** = 213)	***p-***value	21-34 (***n*** = 224)	> 34 (***n*** = 214)	***p***-value	Medical only (***n*** = 113)	Medical + recreational (***n*** = 326)	***p***-value	
Never received information	102 (48.57)	122 (57.28)	0.07	135 (60.27)	97 (45.33)	0.002	60 (53.10)	172 (52.76)	0.95	232 (52.85)
Complexity of the process	98 (46.67)	69 (32.39)	0.003	96 (42.86)	78 (36.45)	0.17	38 (33.63)	137 (42.02)	0.13	175 (39.86)
Difficulty accessing medical cannabis clinics	61 (29.05)	36 (16.90)	0.003	60 (26.80)	42 (19.63)	0.07	20 (17.70)	82 (25.15)	0.11	102 (23.23)
The delay before obtaining cannabis is too long	43 (20.48)	24 (11.27)	0.009	44 (19.64)	27 (12.62)	0.04	16 (14.16)	55 (16.87)	0.50	71 (16.17)
Higher price of cannabis	35 (16.67)	16 (7.51)	0.004	23 (10.27)	30 (14.02)	0.23	16 (14.16)	37 (11.35)	0.43	53 (12.07)
Poor customer service with the clinics	7 (3.33)	5 (2.35)	0.54	8 (3.57)	6 (2.80)	0.65	7 (6.19)	7 (2.15)	0.03	14 (3.19)
Not satisfied with the characteristics of products	24 (11.43)	10 (4.69)	0.01	17 (7.59)	20 (9.35)	0.51	12 (10.62)	25 (7.67)	0.33	37 (8.43)
The inability to choose the products yourself	41 (19.52)	39 (18.31)	0.75	49 (21.88)	34 (15.89)	0.07	16 (14.16)	67 (20.55)	0.13	83 (18.91)
Follow-up with a doctor does not suit me	45 (21.43)	38 (17.84)	0.35	53 (23.66)	36 (16.82)	0.07	11 (9.73)	78 (23.93)	0.001	89 (20.27)
Refusal of physician	13 (6.19)	22 (10.33)	0.12	14 (6.25)	21 (9.81)	0.16	11 (9.73)	24 (7.36)	0.42	35 (7.97)
Other reasons^c^	21 (10.00)	21 (9.86)	0.96	20 (8.93)	23 (10.75)	0.52	15 (13.27)	28 (8.59)	0.15	43 (9.79)

Comparisons between groups were made using chi-square tests and *p*-value calculated. A *p*-value <0.05 was considered as statistically significant

^a^Sixteen participants who reported other gender were not included in the stratification

^b^Fifty participants whose answer was “I don’t know” were not considered in the denominator, in addition to one missing for age categorization

^c^Other reasons included the following: treated condition does not require a medical prescription (minor symptoms), in the process to obtain medical cannabis; no prescription, combine medical access with SQDC access, never engaged in the process/does not feel the need for a medical prescription, stigmatization by the healthcare system, legal access at SQDC thus no need to go in clinics, no clinic near their location

### Treated health conditions

The number of treated conditions per participant varied from 1 to 13. Only 13% reported using cannabis to treat a single health condition or symptom. About two-thirds (65.64%) of respondents were using cannabis for 2 to 5 conditions or symptoms while 20.99% treated 6 or more conditions. Cannabis was more frequently used to treat psychological conditions than physical conditions (85.57% vs 74.43%). Anxiety (70.93%), insomnia (56.49%), depression (37.94%), and attention deficit hyperactivity disorder (ADHD) (25.57%) were the most frequently reported psychological conditions. Chronic non-cancer pain (53.40%), headache/migraine (28.45%), muscle spasticity (16.70%), and bowel disease (11.34%) were the most reported physical conditions. Significant differences between men and women (*p*-value < 0.05) were observed for few conditions. Indeed, men were more likely to report treating ADHD and shyness while women were likely to report treating nausea (Table 3). The use of cannabis for other reported conditions including chronic pain was similar between men and women (*p*-value > 0.05).

**Table 3:** Conditions and symptoms treated with cannabis according to gender and age among individuals who reported self-medication with cannabis in an online survey in Quebec from November 2020 to January 2021 (*n* = 489)

Treated conditions	Gender (missing = 3; 16 participants with “other gender” not stratified)			Age (years) (missing = 4)			Total
	Men (***n*** = 233)	Women (***n*** = 237)	***p***-value	≤ 34 (***n*** = 251)	> 34 (***n*** = 234)	***p***-value	
**Psychological disorders**	199 (85.41)	202 (85.23)	0.96	226 (90.04)	189 (80.77)	0.0037	415 (85.57)
Anxiety	155 (66.52)	176 (74.26)	0.06	196 (78.09)	148 (63.253)	0.0003	344 (70.93)
Depression	95 (40.77)	78 (32.91)	0.07	113 (45.02)	71 (30.34)	0.0009	184 (37.94)
Insomnia	136 (58.37)	129 (54.43)	0.39	148 (58.96)	126 (53.85)	0.25	274 (56.49)
PTSD^a^	33 (14.16)	40 (16.88)	0.41	43 (17.13)	34 (14.53)	0.43	77 (15.88)
ADHD^b^	75 (32.19)	47 (19.83)	0.002	68 (27.09)	56 (23.93)	0.42	124 (25.57)
Shyness	38 (16.31)	14 (5.91)	0.0003	43 (17.13)	11 (4.70)	< 0.0001	54 (11.13)
**Somatic disorders**	168 (72.10)	184 (77.64)	0.17	179 (71.31)	182 (77.78)	0.103	361 (74.43)
Chronic non-cancer pain	126 (54.08)	126 (53.16)	0.84	115 (46.82)	144 (61.54)	0.0005	259 (53.40)
Headaches/migraines	61 (26.18)	74 (31.22)	0.23	86 (34.26)	52 (22.22)	0.003	138 (28.45)
Bowel disease	22 (9.44)	33 (13.92)	0.13	20 (7.97)	35 (14.96)	0.015	55 (11.34)
Muscle spasticity	47 (20.17)	33 (13.92)	0.07	36 (14.34)	45 (19.23)	0.14	81 (16.70)
Nausea/vomiting (unrelated to chemotherapy)	15 (6.44)	30 (12.66)	0.02	28 (11.16)	20 (8.55)	0.33	48 (9.90)
Endometriosis or other gynecological disorder	NA	36 (15.19)	-	27/125 (21.60)	8/112 (7.14)	0.0017	35/237 (14.77)
**Unclassified**							
Loss of appetite	46 (19.74)	41 (17.30)	0.50	57 (22.71)	35 (14.96)	0.029	92 (18.97)
Sexual disorder	14 (6.01)	19 (8.02)	0.39	20 (7.97)	13 (5.56)	0.29	33 (6.80)
Other conditions^c^	45 (19.31)	32 (13.50)	0.09	31 (12.35)	50 (21.37)	0.0078	81 (16.70)

Comparisons between groups were made using chi-square tests and *p*-value calculated. A *p*-value < 0.05 was considered as statistically significant

^a^*PTSD* Post-traumatic stress disorder

^b^*ADHD* Attention deficit hyperactivity disorder

^c^Other conditions include cancer pain (*n* = 3), cancer (*n* = 7), palliative care (*n* = 1), schizophrenia/psychosis (*n* = 3), nausea due to chemotherapy, obesity (*n* = 7), diabetes (*n* = 8), weight loss (*n* = 12), alcohol or opioid withdrawal (*n* = 15), tic disorder/Gilles de la Tourette’s syndrome (*n* = 3), tremor/Parkinson disease (*n* = 7), bladder disorder/incontinence (*n* = 8), asthma (*n* = 1), autism (*n* = 1), congestion (*n* = 1), trigger finger (*n* = 1), gender dysphoria (*n* = 1), rash (*n* = 1), nicotine withdrawal symptom (*n* = 2), hypothyroidism (*n* = 1), snoring (*n* = 1), chronic obstructive pulmonary disease (*n* = 1), psoriasis (*n* = 10), Ehlers-Danlos syndrome (*n* = 1), restless legs syndrome (*n* = 3), borderline personality disorder (*n* = 2), shingles (*n* = 1), suicidal ideation (*n* = 1), Meniere’s disease (*n* = ), itchy skin (*n* = 1), mental/ocular fatigue (*n* = 1)

Conditions treated also slightly varied by age as presented in Table 3. Participants under 34 years treat psychological conditions more often than older participants (90.04% vs 80.77%, *p* = 0.0037) (Table 3).

### Patterns of cannabis use

Table 4 displays the reported patterns of cannabis use, with stratification for gender, age, and the type of use (exclusively medical or mixed). Regarding the THC and CBD ratio, 35% used products with higher THC than CBD and 13% used products with only THC. Regarding potency, about two-thirds reported they mostly used products with THC concentration > 10% while 45% used products with CBD concentration > 10%. Few differences in the patterns of use were observed according to the type of cannabis use (Table 4 and Supplemental Table 1). For example, the pairwise comparisons showed that there was significantly more use of products with equal THC/CBD and products with THC > CBD versus products with only CBD in mixed cannabis users than in exclusive medical users. Mixed cannabis users also significantly use more products with THC concentration > 20% versus < 1% compared to exclusive medical users. Younger individuals (< 34 years) were more likely to use products with 1–10% CBD versus products with CBD > 20% compared to older individuals. Men were more likely to use products with THC >20% versus THC <1% than women (Table 4 and Supplemental Table 1).

**Table 4:** Description of cannabis products used for self-treatment and mode of administration, stratified according to the type of cannabis use among individuals who reported self-medication with cannabis in an online survey in Quebec from November 2020 to January 2021 (*n* = 489)

	Gender (16 participants with “other gender” not stratified)			Age (years)			Type of cannabis use			Total
	Men	Women	***p***-value^**$**^	≤ 34	>34	***p***-value^**$**^	Medical use only	Recreational + medical use	***p***-value^**$**^	
**THC and CBD ratio**	*n* = 231	***n*= 229**	0.0150	*n* = 245	*n* = 230	0.9979	*n* = 120	*n* = 356	<0.0001	*n* = 476
Equal THC and CBD	50 (21.65)	36 (15.72)		46 (18.78)	43 (18.70)		16 (13.33)	73 (20.51)		89 (18.70)
CBD > THC	39 (16.88)	56 (24.45)		49 (20.00)	47 (20.43)		33 (27.50)	63 (17.70)		96 (20.17)
THC > CBD	85 (36.80)	75 (32.75)		86 (35.10)	83 (36.09)		33 (27.50)	136 (38.20)		169 (35.50)
Only CBD	21 (9.09)	37 (16.16)		31 (12.65)	28 (12.17)		27 (22.50)	32 (8.99)		59 (12.39)
Only THC	36 (15.58)	25 (10.92)		33 (13.47)	29 (12.61)		11 (9.17)	52 (14.61)		63 (13.24)
**CBD concentration**	*n* = 217	*n* = 216	0.5007	*n* = 229	*n* = 219	0.0028	*n* = 110	*n* = 338	< 0.0001	*n* = 448
<1%	40 (18.43)	31 (14.35)		41 (17.90)	33 (15.07)		12 (10.91)	62 (18.34)		74 (16.52)
1–10%	82 (37.79)	78 (36.11)		95 (41.48)	73 (33.33)		30 17.86)	138 (40.83)		168 (37.50)
> 10–20%	61 (28.11)	64 (29.63)		68 (29.69)	60 (27.40)		28 (25.45)	100 (29.59)		128 (28.57)
>20%	34 (15.67)	43 (19.91)		25 (10.92)	53 (24.20)		40 (36.36)	38 (11.24)		78 (17.41)
**THC concentration**	*n* = 228	*n* = 226	<.0001	*n* = 243	*n* = 226	0.0764	*n* = 115	*n* = 355	<0.0001	*n* = 470
<1%	23 (10.09)	43 (19.03)		32 (13.17)	35 (15.49)		33 (28.70)	34 (9.58)		67 (14.26)
1–10%	37 (16.23)	60 (26.55)		55 (22.63)	46 (20.35)		29 (25.22)	72 (20.28)		101 (21.49)
>10–20%	73 (32.02)	71 (31.42)		89 (36.63)	62 (27.43)		18 (15.65)	133 (37.46)		151 (32.13)
>20%	95 (41.67)	52 (23.01)		67 (27.57)	83 (36.73)		35 (30.43)	116 (32.68)		151 (32.13)
**Cannabis strain**	*n* = 214	*n* = 196	0.3395	*n* = 222	*n* = 203	0.6900	*n* = 88	*n* = 338	0.8550	*n* = 426
Sativa	47 (21.96)	50 (25.51)		48 (21.62)	51 (25.12)		21 (23.86)	79 (23.37)		100 (23.47)
Indica	63 (29.44)	60 (30.61)		70 (31.53)	56 (27.59)		29 (32.95)	97 (28.70)		126 (29.58)
Hybrid	49 (22.90)	50 (25.51)		52 (23.42)	52 (25.62)		20 (22.73)	84 (24.85)		104 (24.41)
Mixed cannabis	55 (25.70)	36 (18.37)		52 (23.42)	44 (21.67)		18 (20.45)	78 (23.08)		96 (22.54)
**Route of administration** (*n* = 489)^a^				*n* = 252	*n* = 236		*N* = 122	*N* = 367		*n* = 489
Smoking	193 (82.13)	188 (78.89)	0.3889	217 (86.11)	179 (75.85)	0.0038	76 (62.30)	321 (87.47)	<.0001	397 (81.19)
Vaping	52 (22.13)	34 (14.29)	0.0270	48 (19.05)	38 (16.10)	0.3934	17 (13.93)	70 (19.07)	0.1985	87 (17.79)
Vaporizing^b^	67 (28.51)	63 (26.47)	0.6193	71 (28.17)	63 (26.69)	0.7144	40 (32.79)	95 (25.89)	0.1396	135 (27.61)
Oral administration	115 (48.94)	114 (47.90)	0.8215	123 (48.81)	112 (47.46)	0.7652	60 (49.18)	176 (47.96)	0.8147	236 (48.26)
**Frequency of use**	*n* = 220	*n* = 220	0.0539	*n* = 237	*n* = 217	0.4489	*N* = 114	*N* = 341	0.0068	*n* = 455
< 1 day/month	8 (3.64)	18 (8.18)		15 (6.33)	11 (5.07)		13 (11.40)	13 (3.81)		26 (5.71)
1 day/month	6 (2.73)	13 (5.91)		14 (5.91)	5 (2.30)		3 (2.63)	16 (4.69)		19 (4.18)
2–3 days/month	22 (10.00)	31 (14.09)		26 (10.97)	28 (12.90)		18 (15.79)	36 (10.56)		54 (11.87)
1–2 days/week	21 (9.55)	23 (10.45)		24 (10.13)	21 (9.68)		13 (11.40)	33 (9.68)		46 (10.11)
3–4 days/week	34 (15.45)	23 (10.45)		32 (13.50)	25 (11.52)		18 (15.79)	39 (11.44)		57 (12.53)
5–6 days/week	19 (8.64)	22 (10.00)		20 (8.44)	25 (11.52)		7 (6.14)	38 (11.14)		45 (9.89)
Daily	110 (50.00)	90 (40.91)		106 (44.73)	102 (47.00)		42 (36.84)	166 (48.68)		208 (45.71)
**Duration of use for medical purpose**	*n* = 235	*n* = 238	0.1264	*n* = 252	*n* = 236	0.2320	*N* = 122	*N* = 367	< 0.0001	*n* = 489
< 1 month	4 (1.70)	8 (3.36)		8 (3.17)	4 (1.69)		6 (4.92)	6 (1.63)		12 (2.45)
1 to 6 months	15 (6.38)	25 (10.50)		19 (7.54)	23 (9.75)		15 (12.30)	27 (7.36)		42 (8.59)
6–12 months	31 (13.19)	42 (17.65)		46 (18.25)	28 (11.86)		25 (20.49)	49 (13.35)		74 (15.13)
1 to 2 years	45 (19.15)	44 (18.49)		45 (17.86)	46 (19.49)		32 (26.23)	59 (16.08)		91 (18.61)
> 2 years	140 (59.68)	119 (50.00)		134 (53.17)	135 (57.20)		44 (36.07)	226 (61.58)		270 (55.21)

Comparisons between groups were made using chi-square tests and *p*-value calculated. A *p*-value <0.05 was considered as statistically significant

*THC* Tetrahydrocannabinol, *CBD* Cannabidiol

^$^See supplemental Table 1 for pairwise comparisons for categorical variables with a significant global *p*-value

^a^As routes of administration were not mutually exclusive, a global *p*-value was not computed (the comparisons were made for each single route of administration)

^b^Vaporizing consists to heat dry cannabis to release active ingredients as opposed to vaping that uses liquid preparation

Regarding modes of use, more than one mode of use was reported by some participants. Smoking was reported by the majority of participants (81.19%) followed by oral administration (48.26%) (Table 4). Smoking was less preferred by exclusive medical users compared to mixed users (Table 4). Young participants were more likely to smoke their cannabis.

For frequency of use, 45.71% of respondents reported daily cannabis use while 32.53% reported weekly to near-daily use.

The majority of participants reported treating a health condition with cannabis for ≥ 1 year.

### Concurrent use of other medications

A total of 276 (56.44%) participants reported having other prescribed drugs. Among them, 223 reported using their prescribed drugs while 53 mentioned not using their drugs. Drugs’ names were provided by 247 participants. The most mentioned drugs were for pain and psychiatric disorders (Table 5).

**Table 5:** Classes of other prescribed or used drugs reported by a subset of study participants who reported self-medication with cannabis in an online survey in Quebec from November 2020 to January 2021 (*n* = 247)

Likely indication	Drug class	Users (***n*** = 247, 50.51%)
Pain	Unspecified analgesic	36 (14.57)
	Opioids	20 (8.10)
	NSAID	46 (18.62)
	Coanalgesic	17 (6.88)
	Muscle relaxant	21 (8.50)
	Anti-migraine	5 (2.02)
	Corticosteroid	2 (0.81)
	Intestinal anti-inflammatory drug (e.g., mesalamine)	2 (0.81)
Mood/anxiety/sleep disorder	Antidepressant	84 (34.01)
	Serotonin-norepinephrine reuptake inhibitor	4 (1.62)
	Anxiolytic	30 (12.15)
	Hypnotic (z-class or benzodiazepine)	34 (13.77)
	Antipsychotic	27 (10.93)
	Mood stabilizers	4 (1.62)
Attention deficit hyperactivity disorder	Psychostimulant	27 (10.93)
Cardio-vascular/metabolic/digestive	Antidiabetic	2 (0.81)
	Antiacid	10 (4.05)
	Antihypertensive	5 (2.02)
	Anticoagulant	1 (0.40)
	Beta-blocker	5 (2.02)
Asthma/smoking/allergies	Antihistaminic	2 (0.81)
	Nicotine replacement therapy	1 (0.40)
	Inhaled corticosteroid	2 (0.81)
Others	Oral contraceptive	2 (0.81)
	Chemotherapy	1 (0.40)
	Natural health product	9 (3.64)
	Others	16 (6.48)

Co-analgesics include gabapentinoid or tricyclic antidepressants

*NSAID* Nonsteroidal anti-inflammatory drugs

### Healthcare resources and other resources utilization

About 46% of study participants reported that all their treated conditions were diagnosed by a physician, with a higher representation of exclusive medical cannabis users in this category (60.83% versus 41.16% for mixed users) (Table 6).

**Table 6:** Healthcare resources and other resources utilization by individuals who reported self-medication with cannabis in an online survey in Quebec from November 2020 to January 2021 (*n* = 489)

	Gender			Age (years)			Type of cannabis use			Total participants (***n*** = 489) ***N*** (%)^**a**^
	Men	Women	***p***-value^**$**^	≤34	>34	***p***-value^**$**^	Medical use only (***n*** = 122)	Medical + recreational use (***n*** = 367)	***p***-value^**$**^	
Treated condition(s) were diagnosed	***N*= 232**	***N*= 234**	0.050	***N*= 248**	***N*= 233**	**<.0001**	***N*= 120**	***N*= 362**	0.0009	***N*= 482**
Yes, all treated conditions	95 (40.95)	122 (52.14)		86 (34.68)	135 (57.94)		73 (60.83)	149 (41.16)		222 (46.06)
Yes, but not all treated conditions	85 (36.64)	72 (30.77)		97 (39.11)	67 (28.76)		29 (24.17)	135 (37.29)		164 (34.02)
No	52 (22.41)	40 (17.09)		65 (26.21)	31 (13.30)		18 (15.00)	78 (21.55)		96 (19.92)
Declaration of cannabis use to healthcare professionals^c^	*N* = 191	*N* = 151	0.1345	*N* = 170	*N* = 180	0.0276	*N* = 90	*N* = 261	0.034	***N*= 351**
Yes, always	111 (58.12)	72 (47.68)		78 (45.88)	107 (59.44)		51 (56.67)	135 (51.72)		186 (52.99)
Yes, sometimes	56 (29.32)	52 (34.44)		65 (38.24)	47 (26.11)		20 (22.22)	92 (35.25)		112 (31.91)
No	24 (12.57)	27 (17.88)		27 (15.88)	26 (14.44)		19 (21.11)	34 (13.03)		53 (15.10)
Resources consulted for cannabis use	*N* = 235	*N* = 238		*N* = 252	*N* = 236		*N* = 122	*N* = 367		*N* = 489
Physician	87 (37.02)	51 (21.43)	0.0002	67 (26.59)	75 (31.78)	0.2070	34 (27.87)	109 (29.70)	0.700	143 (29.24)
Pharmacist	19 (8.09)	17 (7.14)	0.70	21 (8.33)	15 (6.36)	0.4037	9 (7.38)	27 (7.36)	0.99	36 (7.36)
Legal recreational cannabis store agent	73 (31.06)	102 (42.86)	0.0079	85 (33.72)	95 (40.25)	0.1355	53 (43.44)	127 (34.60)	0.079	180 (36.81)
Illegal seller	25 (10.64)	18 (7.56)	0.24	27 (10.71)	18 (7.63)	0.2388	6 (4.92)	39 (10.63)	0.059	45 (9.20)
Agent from a cannabis production company	18 (7.66)	13 (5.46)	0.33	15 (5.95)	17 (7.20)	0.5769	10 (8.20)	22 (5.99)	0.39	32 (6.54)
Psychologist or psychotherapist	26 (11.06)	15 (6.30)	0.066	25 (9.92)	16 (6.78)	0.2113	9 (7.38)	32 (8.72)	0.64	41 (8.38)
Naturopath or herborist	9 (3.83)	6 (2.52)	0.42	9 (3.57)	7 (2.97)	0.7075	4 (3.28)	12 (3.27)	0.99	16 (3.27)
Other resources^a^	31 (13.19)	20 (8.40)	0.093	24 (9.52)	31 (13.14)	0.2074	19 (15.57)	36 (9.81)	0.081	55 (11.25)
Never consulted	88 (37.45)	98 (41.18)	0.406	108 (42.86)	83 (35.17)	0.0821	39 (31.97)	152 (41.42)	0.064	191 (39.06)
Information sought by consulting a resource (*n* = 298 as 191 never consulted a resource)	*N* = 147	*N* = 140		*N* = 144	*N* = 153		*N* = 83	*N* = 215		*N* = 298
Methods of cannabis consumption	28 (19.05)	45 (32.14)	0.011	36 (25.00)	38 (24.84)	0.9740	28 (33.73)	46 (21.40)	0.027	74 (24.83)
Available varieties and concentrations/potencies of cannabis products	69 (46.94)	85 (60.71)	0.027	78 (54.17)	84 (54.90)	0.8988	47 (56.63)	115(53.49)	0.626	162 (54.36)
Efficacy of cannabis	78 (53.06)	86 (61.43)	0.152	85 (59.03)	85 (55.56)	0.5455	52 (62.65)	118 (54.88)	0.225	170 (57.05)
Safety of cannabis/adverse effects	31 (21.09)	24 (17.14)	0.396	35 (24.31)	22 (14.38)	0.0299	17 (20.48)	41 (19.07)	0.783	58 (19.46)
Safety to combine cannabis with other medication	35 (23.81)	37 (26.43)	0.609	36 (25.00)	40 (26.14)	0.8214	25 (30.12)	51 (23.72)	0.256	76 (25.50)
Subjective effect of cannabis (‘high’)	33 (22.45)	23 (16.43)	0.198	42 (29.17)	18 (11.76)	0.0002	4 (4.82)	56 (26.05)	<0.0001	60 (20.13)
Other^b^	12 (8.16)	5 (3.57)	0.099	12 (8.33)	8 (5.23)	0.2860	4 (4.82)	16 (7.44)	0.417	20 (6.71)
Would like to have access to a healthcare professional regarding medical use of cannabis (*n* = 343; missing = 14)^c^										
Yes	146 (79.78)	122 (80.79)	0.82	136 (82.93)	139 (78.09)	0.2602	72 (80.00)	204 (80.63)	0.90	276 (80.47)

Comparisons between groups were made using chi-square tests and *p*-value calculated. A *p*-value <0.05 was considered as statistically significant

^$^See Supplemental Table 1 for pairwise comparisons for categorical variables with a significant global *p*-value

^a^Other resources included friends/relative/other patients; Internet (Health Canada documentation, scientific articles, online forums), nurses, social worker, physiotherapist, and nutritionist

^b^Other included substitution of cannabis with benzodiazepines and antidepressants, information on the medical access process, if their health condition could be treated with cannabis, risk of dependence with cannabis, information on the reimbursement by insurance, information on the endocannabinoid system, what to do in case of intoxication

^c^These variables were completed by a subset of the participants (*n* = 357) as these questions were added to the questionnaire after the start of the survey

For information on cannabis, participants reported having consulted SQDC retailers (36.81%), physicians (29.24%), illegal sellers (9.20%), and pharmacists (7.36%). However, 39% of all participants reported that they had never consulted a resource about their self-medication with cannabis (Table 6).

Specifically focusing on the 36 participants who reported having consulted a pharmacist, the main reason was to ask for the safety to combine cannabis with other medications (29/36). The satisfaction with the pharmacist’s advice was reported by 19/36 (Supplemental Table 2).

The three questions added after the launch of the survey were completed by 351 participants. About 15% of them reported that they never declared their self-medication with cannabis to healthcare professionals while 32% reported that they sometimes declared their cannabis use (Table 6). No significant differences were observed when the results were stratified according to the type of cannabis use, gender, or age.

Interestingly, 80.47% of all respondents answered that they would like to have access to healthcare professionals for advice related to their medical use of cannabis.

### Impact of COVID-19 on cannabis consumption

From the 357 participants who completed the three questions added after the start of the survey, 18 indicated that the question on the pandemic effect did not apply to them (the question was addressed to participants who were self-medicating with cannabis before the pandemic). From the remaining 339 participants, 139 (41.00%), 185 (54.57%), and 15 (4.43%) reported an increase, no change, and a decrease in their use of cannabis, respectively.

## Discussion

This study suggests that individuals who self-medicate with cannabis are more likely to be young, and use cannabis to treat a wide range of conditions. The reported reasons for self-medication include the lack of information as well as administrative and medical issues. The patterns of cannabis use were highly variable among the study participants, and more than half reported the use of other medications. Finally, not all participants systematically report their cannabis use to healthcare providers. Collectively, these descriptive data provide a complete picture of self-medication with cannabis that can contribute to understanding the problem. To our knowledge, this is the first study that formally described self-medication with cannabis in the general population post-recreational cannabis legalization in Canada.

The observation that individuals who self-medicate with cannabis are mainly young is consistent with the portrait of cannabis use in the general population (Statistics Canada 2020; Roy and Conus 2020). Our recruitment methods (online survey with a focus on social media) may in part explain this higher representativity of younger individuals in our sample. Moreover, mixed medical and recreational users tended to be older than exclusive medical users in our study (73% of medical + recreational users were ≤ 40 years versus 53% for exclusive medical users). A survey conducted before the legalization of recreational cannabis in Canada showed a similar portrait (mean age was 31.1 years for recreational + medical users versus 40.7 years for exclusive medical users) (Turna et al. 2020). In a recent study that included young US participants (aged 18–25 years) with hazardous cannabis use, older individuals were more likely to self-medicate their pain with cannabis (Wallis et al. 2022). In our survey, the observed similar proportion of men and women is not consistent with previous surveys of cannabis users (Turna et al. 2020; Lucas and Walsh 2017). For example, Turna et al. observed a higher proportion of women (59%) among cannabis users (exclusive medical and mixed users) who were surveyed before cannabis legalization in Canada (Turna et al. 2020). In the US sample of young adults, females were more likely to report self-medication of anxiety with cannabis than males (Wallis et al. 2022). Another US study of HIV patients also observed that women were more likely to use cannabis for self-medication (Greenwald et al. 2021). However, Sexton et al. observed a higher proportion of men (54%) in another US sample (Sexton et al. 2016). In a 2015 study of patients registered with a licensed cannabis producer (i.e., patients who likely received a medical prescription), Lucas et al. also observed a higher proportion of men (73%) in their sample (Lucas and Walsh 2017). Our method of recruitment (advertisement on social media with an emphasis towards women) and the fact that some of the groups who shared our survey were women associations may explain our similar proportion of men and women. The observation that women in our sample more often report exclusive medical use of cannabis than men (59% women vs 37% men) was also observed by Turna et al. (77% vs 22%) (Turna et al. 2020). As opposed to age, where individuals were mainly younger, it is interesting to note that individuals with low, medium, and high income were all relying on cannabis to self-medicate their pathology (44% of our sample had annual income >50,000$). This observation is consistent with previous studies (Turna et al. 2020; Lucas and Walsh 2017). Indeed, 42% of Ontario individuals who reported medical use of cannabis (either self-medication or via a prescription) had annual income > 60,000$ (Turna et al. 2020). In a US sample of cannabis users for medical reasons, 51% had annual income > 40,000 US dollars (Sexton et al. 2016).

The main conditions treated by participants in our sample are similar to those reported in previous surveys of patients seeking cannabis to treat a health condition (Sexton et al. 2016; Turna et al. 2020; Lucas and Walsh 2017). Indeed, Sexton et al. observed in a US sample of medical cannabis authorized and self-medicated subjects that the main conditions treated with cannabis were pain (61%), anxiety (58%), and depression (50%) (Sexton et al. 2016). In a sample of Eastern Canadian students who reported medication with cannabis (with 83% of self-medication), the main reason for use was to treat mental health issues (Smith et al. 2021). The main conditions treated with cannabis in self-medication in a sample of US young adults were anxiety (82%), sleep disorders (79%), depression (59%), and pain (40%). The use of cannabis to treat these conditions is consistent with the suggested pharmacologic effects of cannabis and cannabinoids on pain and mental health in the literature (Whiting et al. 2015; Montero-Oleas et al. 2020). The high number of conditions treated per participant in our study and in general is problematic and suggests that cannabis may be perceived as effective for a wide range of conditions, which is not supported by current literature. More communication is needed to inform patients on the lack of scientific evidence on the efficacy of cannabis for most of the claimed indications, the potential risks, the potential interactions with other drugs, and the necessity of an evaluation and follow-up with a healthcare professional to minimize potential risks.

Regarding patterns of use, the observation that THC concentration >20% was reported by a high proportion of participants (i.e., 32%) is a concern as it may expose users to acute toxic effects as well as to long-term adverse effects of cannabis (Health Canada n.d.). This is particularly a concern as a majority of participants reported using cannabis for longer than 1 year to treat a health condition (74%). The lack of medical assistance may explain this use of high concentrations of THC. In previous studies of patients who self-medicate a health condition with cannabis, proportions of patients who reported the use of products with high doses of THC were also high (Sexton et al. 2016; Turna et al. 2020; Stueber and Cuttler 2022). In a study of students who mostly use cannabis to self-medicate their ADHD symptoms, the use of products with high THC/low CBD was reported by 41% of participants while 24% reported use of high THC/high CBD products (Stueber and Cuttler 2022). The use of products with THC concentration > 20% was reported by 27% of this study’s participants (Stueber and Cuttler 2022). Turna et al. also observed in a Canadian sample that products with high THC concentration were reported to be used by 32% of the study participants, with the highest proportion observed among mixed cannabis users (Turna et al. 2020). In a US study of individuals who self-medicate with cannabis, 45.8% of participants mentioned the “claims of high delta 9-tetrahydrocannabinol (THC) potency” as one of the top factors to select their cannabis products (Sexton et al. 2016).

Consistent with the fact that CBD is usually presented as having therapeutic benefits but has less or no psychoactive effects as compared to THC (Health Canada n.d.; MacCallum and Russo 2018), we observed that exclusive medical users had a preference for CBD-dominant products. This preference for CBD-dominant products was previously observed by Turna et al. (35% of exclusive medical users versus 10% of mixed users reported using products with low THC/high CBD concentration (Turna et al. 2020). In most of the previous studies of self-medication with cannabis, information regarding the doses or ratios of THC and CBD was not provided to allow further comparisons with our data (Wallis et al. 2022; Greenwald et al. 2021; Osborn et al. 2015; Sinclair et al. 2020; Hansen et al. 2020).

As compared to previous studies of medical cannabis users (Sexton et al. 2016; Turna et al. 2020; Lucas and Walsh 2017), smoking was the preferred consumption method in our sample. This chronic smoking of cannabis (a majority of participants reported using cannabis for more than 1 year) could potentially negatively impact their respiratory health (Gates et al. 2014). In a sample of Australian women who use cannabis as a self-management strategy of endometriosis, exclusively smoking was reported by 50% of participants while 24% reported multiple methods of use including smoking (Sinclair et al. 2020).

Regarding the frequency of cannabis use, almost half of our study participants reported a daily use of cannabis (45%) with 36% of exclusive medical cannabis users and 48% of mixed users. This pattern of use was quite similar to finding from other studies. Indeed, Turna et al. observed that 40% of their study participants reported daily use of cannabis with mixed users more often reporting daily use (42%) than exclusive medical users (32%) (Turna et al. 2020). A similar proportion of daily cannabis users (43.7%) was also observed in a previous study of Australian women who use cannabis to self-manage endometriosis (Sinclair et al. 2020).

The barriers to accessing medical cannabis are consistent with those reported in previous studies (Lucas and Walsh 2017; Valencia et al. 2017; Sznitman and Lewis 2018). However, the main reason in these studies was usually the cost (Lucas and Walsh 2017; Valencia et al. 2017; Sznitman and Lewis 2018), while the lack of information and the complexity of the process were more prevalent in our sample. The fact that our sample specifically targeted self-medicated individuals may explain this difference. Interestingly, the high majority of participants in our study (80%) reported that they would be interested to have counseling and advice from a healthcare professional for their cannabis use. This information suggests that policies and interventions implemented to target those patients for safer use of cannabis within the healthcare system would be successful. More specifically, addressing the reported barriers to accessing cannabis through the medical circuit is warranted. Particularly, adding medical cannabis services in ambulatory care and decomplexifying the process to obtain medical cannabis and communication could help to reduce self-medication with cannabis and contribute to patients’ safety. Developing and strengthening the cannabis-related expertise of healthcare professionals is also needed for them to engage in appropriate and reassuring discussions with their patients on cannabis, rather than strict refusal or stigmatization, as reported by some participants in our survey and in previous surveys (Lucas and Walsh 2017; Valencia et al. 2017). This is particularly important to avoid conveying patients towards recreational cannabis sellers or illegal sellers to seek advice to use cannabis for their medical conditions as observed in this survey.

The proportion of individuals not reporting their cannabis use to the healthcare professionals (15%) was much lower than what was observed in a study of Eastern Canadian postsecondary students before the legalization of recreational cannabis (Smith et al. 2021). In this latter study, up to 60% of participants reported not declaring their cannabis use to healthcare professionals. Because cannabis use was prohibited during this study and the fact that the sample was exclusively made of students as opposed to our study that included participants from the general population may explain the observed difference between the two measures. However, it is important to note that 31% of our study participants reported that they sometimes declare their cannabis use, a proportion that may contain individuals who never report.

### Limitations

First, our study made use of a self-selected convenience sample who mainly accessed the survey from social media. Therefore, the study sample may not be representative of the general population of individuals using recreational cannabis for self-medication. There is also a lack of ethnic diversity. However, this situation is also observed in a previous study that assessed self-medication with cannabis in a US sample (85% were Caucasians versus 93% in our study) (Osborn et al. 2015). Another limitation is the inclusion of only individuals who speak French. Although this population also includes individuals who are bilingual (French and English speakers or French and other language speakers), it may limit the application of the results to individuals who do not speak French. Recall and memory biases could also have affected the accuracy of the results. Social desirability bias could also be a concern for certain questions. However, the use of an anonymized online survey reduced this possibility. Finally, information bias due to the missing data could be a concern. However, the proportion of individuals with missing data per variable was small in general, thus minimizing this issue.

## Conclusion

Despite the lack of clear and strong evidence supporting the health benefits and safety of cannabis, our study shows that cannabis is used to treat a wide range of conditions without a medical prescription or supervision. The use of high doses of THC and CBD and smoking as a preferred method of use may pose certain risks to users. Concerns also exist with the fact that many users do not systematically declare their use to healthcare professionals. Cannabis-prescribed drug interaction is also a concern. Addressing the reported barriers to cannabis access through the medical system is urgently needed.

## Supplementary Information

**Additional file 1: Supplemental Table 1.** Pairwise comparisons with Bonferroni correction of categorical variables in Tables 1, 4 and 6 where the global Chi-Square test *p*-value <0.05. Note: for Bonferroni comparison, the significance level of the p-value is 0.05/number of comparisons per categorical variable.

**Additional file 2: Supplemental Table 2.** Information inquired and satisfaction with the pharmacist for 36 patients who reported having consulted a pharmacist regarding their self-medication use of cannabis, online survey in Quebec from November 2020 to January 2021.

**Additional file 3.** Questionnaire.

## Data Availability

The data are available from the corresponding author (AZ) research institution, i.e., the CHU de Quebec –Université Laval Research Center. Access to the data is contingent to data sharing rules and policies of the research institution.

## Abbreviations

ADHD: Attention deficit hyperactivity disorder
CBD: Cannabidiol
NSAID: Nonsteroidal anti-inflammatory drugs
PTSD: Post-traumatic stress disorder
SQDC: Société Québécoise du cannabis (Quebec public not-for-profit cannabis retail company)
THC: Tetrahydrocannabinol
