# Supplementary material for: A description of self-medication with cannabis among adults with legal access to cannabis in Quebec, Canada
Source: J Cannabis Res. 2022 May 26;4:26. doi: 10.1186/s42238-022-00135-y (PMC9134718; doi:10.1186/s42238-022-00135-y)
Supplement: Supplementary file 1 — Additional file 1: Supplemental Table 1. Pairwise comparisons with Bonferroni correction of categorical variables in Tables 1, 4 and 6 where the global Chi-Square test p-value <0.05. Note: for Bonferroni comparison, the significance level of the p-value is 0.05/number of comparisons per categorical variable. [file 42238_2022_135_MOESM1_ESM.docx]

Supplemental Table 1: Pairwise comparisons with Bonferroni correction of categorical variables in Tables 1, 4 and 6 where the global Chi-Square test p-value <0.05

|  |  |  |  |  |  |  |  |  |
| --- | --- | --- | --- | --- | --- | --- | --- | --- |
| Table 1 variables |  |  |  |  |  |  |  |  |
|  | **Stratification variables in Table 1, 4 or 6** | | | | | | | |
|  | **Gender** | |  | **Age** | |  | **Type of cannabis use** | |
|  | Raw p-value | Bonferroni p-value |  | Raw p-value | Bonferroni p-value |  | Raw p-value | Bonferroni p-value |
| **Variables with a global p-value <0.05 in Table 1** |  |  |  |  |  |  |  |  |
| **Highest level of education** |  |  |  |  |  |  |  |  |
| Primary or High School vs Technical school | 0.6491 | 1.00000 |  |  |  |  |  |  |
| Primary or High School vs college | 0.1286 | 1.00000 |  |  |  |  |  |  |
| Primary or High School vs First cycle university | 0.0027 | 0.02723 |  |  |  |  |  |  |
| Primary or High School vs Second/third cycle University | 0.1497 | 1.00000 |  |  |  |  |  |  |
| Technical school vs college | 0.0388 | 0.38802 |  |  |  |  |  |  |
| Technical school vs First cycle university | 0.0005 | **0.00454** |  |  |  |  |  |  |
| Technical school vs Second/third cycle University | 0.0736 | 0.73590 |  |  |  |  |  |  |
| College vs First cycle university | 0.0697 | 0.69745 |  |  |  |  |  |  |
| College vs Second/third cycle University | 0.6418 | 1.00000 |  |  |  |  |  |  |
| First cycle university vs Second/third cycle University | 0.4400 | 1.00000 |  |  |  |  |  |  |
| **Gender** |  |  |  |  |  |  |  |  |
| Male vs Female |  |  |  |  |  |  | 0.0054 | **0.01625** |
| Male vs Other Gender |  |  |  |  |  |  | 0.9358 | 1.00000 |
| Female vs Other Gender |  |  |  |  |  |  | 0.3134 | 0.94021 |
| **Age (years)** |  |  |  |  |  |  |  |  |
| 21-30 vs 31-40 |  |  |  |  |  |  | 0.0215 | 0.21460 |
| 21-30 vs 41-50 |  |  |  |  |  |  | 0.0148 | 0.14753 |
| 21-30 vs 51-60 |  |  |  |  |  |  | <.0001 | **0.00006** |
| 21-30 vs >60 |  |  |  |  |  |  | <.0001 | **0.00023** |
| 31-40 vs 41-50 |  |  |  |  |  |  | 0.7170 | 1.00000 |
| 31-40 vs 51-60 |  |  |  |  |  |  | 0.0139 | 0.13936 |
| 31-40 vs >60 |  |  |  |  |  |  | 0.0108 | 0.10838 |
| 41-50 vs 51-60 |  |  |  |  |  |  | 0.0494 | 0.49414 |
| 41-50 vs >60 |  |  |  |  |  |  | 0.0301 | 0.30085 |
| 51-60 vs >60 |  |  |  |  |  |  | 0.5642 | 1.00000 |
|  |  |  |  |  |  |  |  |  |
| **Variables with a global p-value <0.05 in Table 4** |  |  |  |  |  |  |  |  |
| **THC and CBD ratio** |  |  |  |  |  |  |  |  |
| Equal THC and CBD vs CBD > THC | 0.0217 | 0.21660 |  |  |  |  | 0.0116 | 0.11557 |
| Equal THC and CBD vs THC > CBD | 0.4510 | 1.00000 |  |  |  |  | 0.7630 | 1.00000 |
| Equal THC and CBD vs Only CBD | 0.0098 | 0.09825 |  |  |  |  | 0.0003 | **0.00267** |
| Equal THC and CBD vs Only THC | 0.9153 | 1.00000 |  |  |  |  | 0.9345 | 1.00000 |
| CBD > THC vs THC > CBD | 0.0622 | 0.62206 |  |  |  |  | 0.0072 | 0.07221 |
| CBD > THC vs Only CBD | 0.5514 | 1.00000 |  |  |  |  | 0.1576 | 1.00000 |
| CBD > THC vs Only THC | 0.0284 | 0.28426 |  |  |  |  | 0.0197 | 0.19711 |
| THC > CBD vs Only CBD | 0.0272 | 0.27211 |  |  |  |  | <.0001 | **0.00081** |
| THC > CBD vs Only THC | 0.4315 | 1.00000 |  |  |  |  | 0.7210 | 1.00000 |
| Only CBD vs Only THC | 0.0128 | 0.12788 |  |  |  |  | 0.0007 | 0.00742 |
| **CBD concentration** |  |  |  |  |  |  |  |  |
| <1% vs 1-10% |  |  |  | 0.8689 | 1.00000 |  | 0.7561 | 1.00000 |
| <1% vs > 10-20% |  |  |  | 0.7541 | 1.00000 |  | 0.3309 | 1.00000 |
| <1% vs >20% |  |  |  | 0.0037 | 0.02214 |  | <.0001 | **0.00003** |
| 1-10% vs 10-20% |  |  |  | 0.5576 | 1.00000 |  | 0.3883 | 1.00000 |
| 1-10% vs >20% |  |  |  | 0.0003 | **0.00209** |  | <.0001 | **0.00000** |
| 10-20% vs >20% |  |  |  | 0.0032 | 0.01918 |  | <.0001 | **0.00008** |
| **THC concentration** |  |  |  |  |  |  |  |  |
| <1% vs 1-10% | 0.6684 | 1.00000 |  |  |  |  | 0.0069 | 0.04140 |
| <1% vs > 10-20% | 0.0324 | 0.19418 |  |  |  |  | <.0001 | **0.00000** |
| <1% vs >20% | <.0001 | **0.00032** |  |  |  |  | 0.0001 | **0.00076** |
| 1-10% vs 10-20% | 0.0551 | 0.33052 |  |  |  |  | 0.0008 | **0.00478** |
| 1-10% vs >20% | <.0001 | **0.00029** |  |  |  |  | 0.3226 | 1.00000 |
| 10-20% vs >20% | 0.0162 | 0.09695 |  |  |  |  | 0.0101 | 0.06073 |
| **Frequency of use** |  |  |  |  |  |  |  |  |
| < 1 day/month vs 1 days/month |  |  |  |  |  |  | 0.0179 | 0.37564 |
| < 1 day/month vs 2-3 days/month |  |  |  |  |  |  | 0.1518 | 1.00000 |
| < 1 day/month vs 1-2 days/week |  |  |  |  |  |  | 0.0651 | 1.00000 |
| < 1 day/month vs 3-4 days/week |  |  |  |  |  |  | 0.1076 | 1.00000 |
| < 1 day/month vs 5-6 days/week |  |  |  |  |  |  | 0.0019 | 0.03950 |
| < 1 day/month vs Daily |  |  |  |  |  |  | 0.0007 | 0.01525 |
| 1 day/month vs 2-3 days/month |  |  |  |  |  |  | 0.1462 | 1.00000 |
| 1 day/month vs 1-2 days/week |  |  |  |  |  |  | 0.2884 | 1.00000 |
| 1 day/month vs 3-4 days/week |  |  |  |  |  |  | 0.1826 | 1.00000 |
| 1 day/month vs 5-6 days/week |  |  |  |  |  |  | 0.9812 | 1.00000 |
| 1 day/month vs Daily |  |  |  |  |  |  | 0.6449 | 1.00000 |
| 2-3 days/month vs 1-2 days/week |  |  |  |  |  |  | 0.5846 | 1.00000 |
| 2-3 days/month vs 3-4 days/week |  |  |  |  |  |  | 0.8436 | 1.00000 |
| 2-3 days/month vs 5-6 days/week |  |  |  |  |  |  | 0.0426 | 0.89532 |
| 2-3 days/month vs Daily |  |  |  |  |  |  | 0.0406 | 0.85247 |
| 1-2 days/week vs 3-4 days/week |  |  |  |  |  |  | 0.7151 | 1.00000 |
| 1-2 days/week vs 5-6 days/week |  |  |  |  |  |  | 0.1434 | 1.00000 |
| 1-2 days/week vs Daily |  |  |  |  |  |  | 0.2292 | 1.00000 |
| 3-4 days/week vs 5-6 days/week |  |  |  |  |  |  | 0.0618 | 1.00000 |
| 3-4 days/week vs Daily |  |  |  |  |  |  | 0.0688 | 1.00000 |
| 5-6 days/week vs Daily |  |  |  |  |  |  | 0.4754 | 1.00000 |
| **Duration of use for medical purpose** |  |  |  |  |  |  |  |  |
| < 1 month vs 1 to 6 months |  |  |  |  |  |  | 0.3706 | 1.00000 |
| < 1 month vs 6-12 months |  |  |  |  |  |  | 0.2778 | 1.00000 |
| < 1 month vs 1 to 2 years |  |  |  |  |  |  | 0.3168 | 1.00000 |
| < 1 month vs > 2 years |  |  |  |  |  |  | 0.0028 | 0.02779 |
| 1 to 6 months vs 6-12 months |  |  |  |  |  |  | 0.8335 | 1.00000 |
| 1 to 6 months vs 1 to 2 years |  |  |  |  |  |  | 0.9509 | 1.00000 |
| 1 to 6 months vs > 2 years |  |  |  |  |  |  | 0.0028 | 0.02794 |
| 6-12 months vs 1 to 2 years |  |  |  |  |  |  | 0.8528 | 1.00000 |
| 6-12 months vs > 2 years |  |  |  |  |  |  | 0.0009 | 0.00874 |
| 1 to 2 years vs > 2 years |  |  |  |  |  |  | 0.0001 | **0.00134** |
| **Variables with a global p-value <0.05 in Table 6** |  |  |  |  |  |  |  |  |
| **Treated condition(s) were diagnosed** |  |  |  |  |  |  |  |  |
| Yes, all treated conditions vs Yes, but not all treated conditions | 0.0478 | 0.14337 |  | <.0001 | **0.00025** |  | 0.0008 | **0.00244** |
| Yes, all treated conditions vs No | 0.0403 | 0.12081 |  | <.0001 | **0.00001** |  | 0.0105 | 0.03141 |
| Yes, but not all treated conditions vs No | 0.7154 | 1.00000 |  | 0.1692 | 0.50755 |  | 0.8292 | 1.00000 |
| **Declaration of cannabis use to healthcare professionals #** |  |  |  |  |  |  |  |  |
| Yes, always vs Yes, sometimes |  |  |  | 0.0080 | 0.02390 |  | 0.0606 | 0.18169 |
| Yes, always vs No |  |  |  | 0.2563 | 0.76896 |  | 0.2342 | 0.70257 |
| Yes, sometimes vs No |  |  |  | 0.3917 | 1.00000 |  | 0.0111 | 0.03325 |

Note: for Bonferroni comparison, the significance level of the p-value is 0.05/number of comparisons per categorical variable
