# Supplementary material for: A description of self-medication with cannabis among adults with legal access to cannabis in Quebec, Canada
Source: J Cannabis Res. 2022 May 26;4:26. doi: 10.1186/s42238-022-00135-y (PMC9134718; doi:10.1186/s42238-022-00135-y)
Supplement: Supplementary file 2 — Additional file 2: Supplemental Table 2. Information inquired and satisfaction with the pharmacist for 36 patients who reported having consulted a pharmacist regarding their self-medication use of cannabis, online survey in Quebec from November 2020 to January 2021. [file 42238_2022_135_MOESM2_ESM.docx]

**Supplemental Table 2: Information inquired and satisfaction with the pharmacist for 36 patients who reported having consulted a pharmacist regarding their self-medication use of cannabis, online survey in Quebec from November 2020 to January 2021**

| Information sought by consulting a pharmacist (n=36) | N |
| --- | --- |
| Advice on the methods of consumption | 3 |
| Advice on the available varieties and concentrations/potencies of cannabis products | 5 |
| Advice on the efficacy of cannabis | 15 |
| Advice on the safety of cannabis | 8 |
| Advice on the safety to combine cannabis with other medication | 29 |
| Satisfaction with the advice from the pharmacist (n=36; missing=4) |  |
| Yes | 19 |
| No | 11 |
| I don’t know | 2 |
| Perceived elements for satisfaction |  |
| The pharmacist referred me to other professional resources (a physician or a cannabis clinic) | 9 |
| I did not perceive a negative opinion from the pharmacist on my cannabis consumption | 13 |
| I felt the pharmacist has negatively judged my consumption of cannabis | 9 |
| The pharmacist verified that cannabis is compatible with my medications | 10 |
| The pharmacist encouraged me to stop or reduce my cannabis consumption | 4 |
| The advice of the pharmacist was beyond my expectation | 1 |
