# Supplementary material for: A description of self-medication with cannabis among adults with legal access to cannabis in Quebec, Canada
Source: J Cannabis Res. 2022 May 26;4:26. doi: 10.1186/s42238-022-00135-y (PMC9134718; doi:10.1186/s42238-022-00135-y)
Supplement: Supplementary file 3 — Additional file 3. Questionnaire. [file 42238_2022_135_MOESM3_ESM.docx]

**ANNEXE 1 - Questionnaire**

**Cheminement de répondants fictifs**

| **Consommateur de cannabis automédication seulement** | **Consommateur de cannabis automédication ET récréatif** | **Consommateur de cannabis récréatif seulement** |
| --- | --- | --- |
| Section 1 - Consommation de cannabis | Section 1 - Consommation de cannabis | Section 1 - Consommation de cannabis |
| Section 2 - Données démographiques | Section 2 - Données démographiques | Section 2 - Données démographiques |
| Section 3 - Usage médical | Section 3 - Usage médical | FIN DU SONDAGE |
| Section 4 - Effets perçus | Section 4 - Effets perçus |  |
| Section 5 - Approvisionnement et encadrement | Section 5 - Approvisionnement et encadrement |  |
| Section 6 - Rôle du pharmacien | Section 6 - Rôle du pharmacien |  |
| FIN DU SONDAGE | FIN DU SONDAGE |  |

Notes :

- Les questions en gris ne sont accessibles que si le répondant a sélectionné une réponse particulière à une question antérieure. Les redirections à partir de ces réponses particulières sont marquées en jaune.
- Le questionnaire comporte 40 questions réparties en 6 sections. Les sections sont identifiées en gris.

___________________________________________________________________________

**Questionnaire du projet de recherche *Qui se soigne à la SQDC ?***

**Début du questionnaire**

Section 1 : Consommation globale de cannabis

1. Au cours des 12 derniers mois, à quelle fréquence avez-vous consommé des produits de cannabis **achetés à la SQDC** ?
   - Je ne consomme pas de cannabis de la SQDC. >>> Sortie du questionnaire
   - Moins de 1 jour par mois
   - 1 jour par mois
   - 2 à 3 jours par mois
   - 1 à 2 jours par semaine
   - 3 à 4 jours par semaine
   - 5 à 6 jours par semaine
   - Une ou plusieurs fois par jour
   - Je consomme du cannabis de la SQDC, mais je ne sais pas à quelle fréquence.
   - Je consomme du cannabis de la SQDC, mais je préfère ne pas indiquer la fréquence de ma consommation.

Mot de remerciement à la sortie du questionnaire : Nous vous remercions du temps que vous avez consacré à cette enquête. Votre participation nous apportera de précieux renseignements pour la réalisation de ce projet de recherche.

1. L’expression **traiter une condition de santé** signifie de soulager un problème médical ou un symptômes (p. ex. de la douleur, de l’insomnie, de l’anxiété, une perte d’appétit, des émotions désagréables, etc.) en utilisant des substances thérapeutiques comme des médicaments prescrits, des médicaments de vente libre ou du cannabis.

L’expression **pour un usage récréatif** signifie de consommer du cannabis pour le plaisir.

Quel(s) usage(s) faites-vous des produits de cannabis **achetés à la SQDC** ?

- - Je consomme pour soulager/traiter un problème de santé ou un symptôme. >>> Continuer vers les autres sections
  - Je consomme pour soulager/traiter un problème de santé ou un symptôme ET pour un usage récréatif. >>> Continuer vers les autres sections + activer question 20
  - Je consomme pour un usage récréatif seulement. >>> Sortie
  - Je ne consomme ni pour soulager/traiter un problème de santé ou un symptôme, ni pour un usage récréatif. >>> Sortie

Section 2 : Caractéristiques démographiques

1. Où avez-vous vu l’invitation à participer à ce questionnaire ?
   - Sur Facebook
   - Dans une pharmacie
   - Par courriel
   - Organismes
   - … (moduler les choix de réponses ultérieurement selon les organismes qui acceptent notre demande)

Veuillez préciser les informations suivantes :

1. Genre :
   - Homme
   - Femme
   - Autre (précisez) :
2. Âge :
   - _______ ans
   - Je préfère ne pas répondre >>> Question 4
3. Dans quelle catégorie d’âge vous situez-vous ?
   - Moins de 15 ans
   - 15 à 17 ans
   - 18 à 20 ans
   - 21 à 24 ans
   - 25 à 34 ans
   - 35 à 54 ans
   - 55 ans et plus
   - Je préfère ne pas répondre
4. Quelle est votre origine ethnique ?
   - Caucasienne
   - Africaine
   - Asiatique
   - Hispanique
   - Autochtone
   - Autre
   - Je ne sais pas
   - Je préfère ne pas répondre
5. Dans quelle région administrative résidez-vous?
   - Abitibi-Témiscamingue
   - Bas-Saint-Laurent
   - Capitale-Nationale
   - Centre-du-Québec
   - Chaudière-Appalaches
   - Côte-Nord
   - Estrie
   - Gaspésie–Îles-de-la-Madeleine
   - Lanaudière
   - Laurentides
   - Laval
   - Mauricie
   - Montérégie
   - Montréal
   - Nord-du-Québec
   - Outaouais
   - Saguenay–Lac-Saint-Jean
6. Dernier niveau de scolarité complété :
   - Études primaires
   - Diplôme d’études secondaires
   - Diplôme d’études professionnelles
   - Diplôme d’études collégiales
   - Diplôme universitaire de premier cycle (ex : baccalauréat, certificat)
   - Diplôme universitaire de cycles supérieurs (ex : maîtrise, doctorat)
   - Non scolarisé
   - Je ne sais pas
   - Je ne préfère ne pas répondre
7. Quel est votre état matrimonial actuel ?
   - Je suis célibataire
   - Je suis en couple (conjoint(e) de fait ou marié(e))
   - Je ne sais pas
   - Je préfère ne pas répondre
8. À combien estimez-vous le revenu de votre ménage ?
   - Moins de 10 000 $
   - De 10 000 $ à moins de 25 000 $
   - De 25 000 $ à moins de 50 000 $
   - De 50 000 $ à moins de 75 000 $
   - De 75 000 $ à moins de 100 000 $
   - De 100 000 $ à moins de 125 000 $
   - De 125 000 $ à moins de 150 000 $
   - Plus de 150 000 $
   - Je ne sais pas
   - Je préfère ne pas répondre

Section 3 : Consommation de cannabis pour traiter une condition de santé

1. Au cours des 12 derniers mois, à quelle fréquence avez-vous consommé du cannabis de la SQDC **aux fins d’autotraitement d’une condition de santé** ?
   - Moins de 1 jour par mois
   - 1 jour par mois
   - 2 à 3 jours par mois
   - 1 à 2 jours par semaine
   - 3 à 4 jours par semaine
   - 5 à 6 jours par semaine
   - Une ou plusieurs fois par jour
   - Je ne sais pas
   - Je préfère ne pas répondre
2. Quelle proportion de votre consommation de cannabis de la SQDC accordez-vous au **traitement de votre condition de santé** ?
   - La totalité du cannabis que je consomme.
   - La majorité du cannabis que je consomme.
   - La moitié du cannabis que je consomme.
   - Moins de la moitié du cannabis que je consomme.
   - Je ne sais pas.
   - Je préfère ne pas répondre.
3. Au cours des 12 derniers mois, avez-vous utilisé les méthodes suivantes pour consommer du cannabis de la SQDC afin de **traiter votre condition de santé** ?

|  | Oui | Non | Ne sais pas |
| --- | --- | --- | --- |
| Je l’ai fumé (joint pré-roulé, fleurs séchées) |  |  |  |
| Je l’ai vapoté (cigarette électronique, vapoteuse) |  |  |  |
| Je l’ai vaporisé (spray, vaporisateur, pouche-pouche, atomiseur) |  |  |  |
| Je l’ai ingéré (pilule, capsule, huile, teinture, concentrés, aliments ou boissons contenant du cannabis) |  |  |  |
| Autre méthode (veuillez préciser) |  |  |  |

1. Quel(s) type(s) de cannabis de la SQDC consommez-vous généralement afin de **traiter votre condition de santé** ?
   - Des produits avec exclusivement du THC
   - Des produits avec plus de THC que de CBD
   - Des produits avec autant de THC que de CBD
   - Des produits avec plus de CBD que de THC
   - Des produits avec exclusivement du CBD
   - Je ne sais pas
   - Je préfère ne pas répondre
2. Au cours des 12 derniers mois, en général, quelle teneur en THC contenaient vos produits de cannabis de la SQDC utilisés pour **traiter votre condition de santé** ?
   - Une teneur faible en THC (moins de 10 % de THC)
   - Une teneur modérée en THC (10 à 20 % de THC)
   - Une teneur élevée en THC (plus de 20 % de THC)
   - Je ne sais pas
   - Je préfère ne pas répondre
3. Au cours des 12 derniers mois, en général, quelle teneur en CBD contenaient vos produits de cannabis de la SQDC utilisés pour **traiter votre condition de santé** ?
   - Une teneur faible en CBD (moins de 10 % de CBD)
   - Une teneur modérée en CBD (10 à 20 % de CBD)
   - Une teneur élevée en CBD (plus de 20 % de CBD)
   - Je ne sais pas
   - Je préfère ne pas répondre
4. Au cours des 12 derniers mois, quelle variété de cannabis de la SQDC avez-vous majoritairement consommé pour **traiter votre condition de santé** ? Sélectionnez toutes les réponses appropriées.
   - Cannabis Sativa
   - Cannabis Indica
   - Cannabis Hybride
   - Cannabis mélangé
   - Je ne sais pas
   - Je préfère ne pas répondre
5. Quels sont les produits du cannabis que vous utilisez actuellement à des fins d’automédication? Veuillez inscrire le nom et la marque des produits dans l’espace ci-dessous.
6. Lorsque vous consommez du cannabis pour des fins récréatives, utilisez-vous les mêmes produits ou des produits avec les mêmes caractéristiques que pour traiter un problème de santé?
   - Oui
   - Non
   - Je ne consomme pas de cannabis à des fins récréatives
   - Je préfère ne pas répondre
7. Quelle(s) problème(s) de santé ou quels symptôme(s) traitez-vous avec du cannabis de la SQDC ? Sélectionnez toutes les réponses qui s’appliquent.

Douleur non cancéreuse (p. ex. arthrite, douleur au dos, hernie, fibromyalgie)

Douleur causée par un cancer

Soins palliatifs

Cancer

Anxiété/stress

Gêne/timidité

Baisse de libido ou autres troubles sexuels

Inattention/Trouble de l’attention avec ou sans hyperactivité (TDA/H)

Dépression (p. ex. déprime, trouble dépressif, maladie bipolaire)

Trouble de stress post-traumatique (TSPT)

Schizophrénie ou psychose

Maux de tête ou migraine

Sclérose en plaques

Nausées ou vomissements dus à la chimiothérapie

Nausées ou vomissements autres que ceux dus à la chimiothérapie

Maladie intestinale (p. ex. maladie de Crohn, colite ulcéreuse, syndrome du côlon irritable)

Raideur musculaire (spasticité ou dystonie)

Insomnie/difficulté d’endormissement

Obésité

Diabète

Épilepsie/convulsions

Perte d’appétit

Perte de poids

Symptômes de sevrage d’alcool ou d’opioïdes

Glaucome

Troubles de tic ou syndrome de Gilles de La Tourette

Tremblements ou Maladie de Parkinson

Démence ou Maladie d’Alzheimer

Problème de vessie/Vessie hyperactive/Incontinence

Autre (veuillez préciser) : __________________________________________

Je ne sais pas

Je préfère ne pas répondre

1. Cette condition de santé a-t-elle été diagnostiquée par un médecin ?
   - Oui
   - Non
   - Je ne sais pas
   - Je préfère ne pas répondre
2. Depuis combien de temps traitez-vous cette (ces) condition(s) de santé avec du cannabis ? Sélectionnez le choix qui estime le mieux cette durée.
   - Champ numérique libre : _______ (indicateur pour choisir si mois ou années)
   - Je ne sais pas
   - Je préfère ne pas répondre
3. Rapportez-vous l'utilisation de cannabis aux professionnels de santé lors de vos visites ou consultations ?
   - Oui, toujours
   - Oui, mais pas toujours
   - Non, jamais
   - Je préfère ne pas répondre
4. Généralement, comment la consommation du cannabis de la SQDC affecte-t-elle votre condition de santé ou vos symptômes ?

Le cannabis aggrave ma condition de santé ou mes symptômes

Le cannabis n’a aucun effet sur ma condition de santé ou mes symptômes

Le cannabis améliore légèrement ma condition de santé ou mes symptômes

Le cannabis améliore modérément ma condition de santé ou mes symptômes

La cannabis améliore grandement ma condition de santé ou mes symptômes

Je ne sais pas

Je préfère ne pas répondre

1. Mis à part le cannabis, utilisez-vous des médicaments prescrits ou non par un médecin pour traiter votre condition de santé ?
   - Oui je prends des médicaments pour cette condition de santé >>> Question 24
   - Non je ne prends aucun médicament pour cette condition de santé
   - Je ne sais pas
   - Je préfère ne pas répondre
2. Dans la mesure du possible, veuillez identifier le nom des médicaments que vous prenez pour la ou les conditions de santé concernées. Si le nom des médicaments vous échappe, veuillez indiquer à quoi sert ce médicament : antidouleur, antiépileptique, sédatif, anxiolytique, etc. _____________________________________________________________________
3. Votre consommation de cannabis de la SQDC pour traiter votre condition de santé a-t-elle eu un impact sur votre utilisation de médicaments ?
   - Oui, les doses de médicaments ont augmenté depuis que je consomme du cannabis.
   - Oui, les doses de médicaments ont diminué depuis que je consomme du cannabis. >>> Question 26
   - Oui, j’ai cessé un ou des médicaments depuis que je consomme du cannabis. >>> Question 26
   - Non, ma médication est demeurée inchangée depuis que je consomme du cannabis.
   - Ne s’applique pas (Je ne consomme aucun médicament.)
   - Je ne sais pas
4. Dans la mesure du possible, veuillez identifier le nom des médicaments que vous avez pu cesser ou diminuer depuis que vous consommez du cannabis pour traiter votre condition. Si le nom des médicaments vous échappe, veuillez indiquer à quoi sert ce médicament : antidouleur, antiépileptique, sédatif, anxiolytique, etc. _____________________________________________________________________

Section 4 : Effets perçus

1. Après avoir consommé du cannabis de la SQDC pour traiter votre condition de santé, ressentez-vous les effets psychologiques suivants ?

|  | Toujours présent | Parfois présent | Jamais présent | Ne sais pas |
| --- | --- | --- | --- | --- |
| Sensation d’euphorie (« high ») |  |  |  |  |
| Sensation de détente (relaxation) |  |  |  |  |
| Augmentation de la libido ou de l’activité sexuelle |  |  |  |  |
| Rires incontrôlables |  |  |  |  |
| Perceptions altérées |  |  |  |  |
| Capacité réduite de réagir rapidement (facultés affaiblies) |  |  |  |  |
| Aiguisement des sens |  |  |  |  |
| Confusion |  |  |  |  |
| Somnolence (fatigue) |  |  |  |  |
| Sensation d’anxiété (nervosité) |  |  |  |  |
| Sensation de panique (peur) |  |  |  |  |
| Hallucinations ou délires (symptômes psychotiques) |  |  |  |  |
| Paranoïa |  |  |  |  |
| Cauchemars |  |  |  |  |
| Autre (veuillez préciser) |  |  |  |  |

1. Ces effets psychologiques ont-ils influencé votre consommation de cannabis de la SQDC ?
   - Oui, ces effets m’ont fait diminuer ma consommation de cannabis
   - Oui, ces effets m’ont fait augmenter ma consommation de cannabis
   - Non, ces effets n’ont pas modifié ma consommation de cannabis
   - Ne s’applique pas
2. Depuis le tout début de votre consommation de cannabis pour traiter votre condition de santé, avez-vous déjà présenté les effets psychologiques suivants ?

|  | Oui souvent | Oui parfois | Non jamais | Ne sais pas |
| --- | --- | --- | --- | --- |
| Difficulté à me concentrer |  |  |  |  |
| Problème de mémoire |  |  |  |  |
| Sensation de déprime (tristesse, dépression) |  |  |  |  |
| Irritabilité |  |  |  |  |
| Diminution de la motivation (apathie) |  |  |  |  |
| Problème d’apprentissage |  |  |  |  |
| Trouble du sommeil |  |  |  |  |
| Trouble anxieux |  |  |  |  |
| Difficulté à contrôler ma consommation de cannabis |  |  |  |  |
| Autre (veuillez préciser) |  |  |  |  |

1. Ces effets psychologiques ont-ils influencé votre consommation de cannabis de la SQDC ?
   - Oui, ces effets m’ont fait diminuer ma consommation de cannabis
   - Oui, ces effets m’ont fait augmenter ma consommation de cannabis
   - Non, ces effets n’ont pas modifié ma consommation de cannabis
   - Ne s’applique pas
2. Après avoir consommé du cannabis de la SQDC pour traiter votre condition de santé, ressentez-vous les effets physiques suivants ?

|  | Toujours présent | Parfois présent | Jamais présent | Ne sais pas |
| --- | --- | --- | --- | --- |
| Sécheresse de la bouche |  |  |  |  |
| Trouble des mouvements ou de coordination |  |  |  |  |
| Faiblesse musculaire |  |  |  |  |
| Difficulté à parler (trouble d’élocution) |  |  |  |  |
| Palpitations (le coeur qui «s’emballe») |  |  |  |  |
| Étourdissements ou vertiges |  |  |  |  |
| Troubles digestifs (nausées, vomissements, diarrhées) |  |  |  |  |
| Tremblements |  |  |  |  |
| Sueurs |  |  |  |  |
| Autre (veuillez préciser) |  |  |  |  |

1. Ces effets physiques ont-ils influencé votre consommation de cannabis de la SQDC ?
   - Oui, ces effets m’ont fait diminuer ma consommation de cannabis
   - Oui, ces effets m’ont fait augmenter ma consommation de cannabis
   - Non, ces effets n’ont pas modifié ma consommation de cannabis
   - Ne s’applique pas
2. Quelle est votre perception de l’effet du cannabis de la SQDC sur votre qualité de vie?

|  | Effet positif | Aucun effet | Effet négatif | Ne sais pas |
| --- | --- | --- | --- | --- |
| Sur ma santé physique (capacité à me déplacer) |  |  |  |  |
| Sur ma santé mentale (humeur, stress concentration, mémoire) |  |  |  |  |
| Sur ma vie de famille ou de couple |  |  |  |  |
| Sur mes relations sociales avec mes amis |  |  |  |  |
| Sur mon travail ou mes études |  |  |  |  |
| Sur mes activités domestiques et mes loisirs |  |  |  |  |
| Sur ma qualité de vie en général |  |  |  |  |

1. Ces effets sur votre qualité de vie ont-ils influencé votre consommation de cannabis de la SQDC ?
   - Oui, ces effets m’ont fait diminuer ma consommation de cannabis
   - Oui, ces effets m’ont fait augmenter ma consommation de cannabis
   - Non, ces effets n’ont pas modifié ma consommation de cannabis
   - Ne s’applique pas

Section 5 : Approvisionnement et encadrement

1. Pour quelle(s) raison(s) n’achetez-vous pas du cannabis sur ordonnance dans une clinique médicale ? Sélectionnez toutes les réponses qui s’appliquent.
   - Le prix du cannabis prescrits ne me convient pas
   - Le service à la clientèle des cliniques de cannabis ne me convient pas
   - Le suivi auprès d’un médecin ne me convient pas
   - La qualité des produits prescrits ne me convient pas
   - La sécurité des produits prescrits ne me convient pas
   - La disponibilité des produits prescrits ne me convient pas
   - Les modes d'administration (p. ex. vaporisateur, huile, herbe séchée, etc.) ne me conviennent pas
   - Les variétés de cannabis sur prescription (p. ex. sativa, indica, etc.) ne me conviennent pas
   - Les arômes et saveurs du cannabis sur prescription ne me conviennent pas
   - La difficulté d’accès aux cliniques médicales de cannabis (succursale, heures d’ouverture)
   - L’impossibilité d’achats en ligne
   - Le délai avant d’obtenir du cannabis prescrit ne me convient pas (temps d’attente ou de livraison)
   - L’impossibilité de choisir soi-même les produits prescrits
2. Avez-vous déjà consulté les ressources suivantes pour obtenir des renseignements ou des conseils à propos de la consommation de produits de cannabis à des fins médicales?

|  | Oui je déjà consulté cette ressource | Non je n’ai jamais consulté cette ressource | Je ne sais pas |
| --- | --- | --- | --- |
| Un conseiller de la SQDC |  |  |  |
| Un conseiller d’une compagnie fabricante de cannabis médical |  |  |  |
| Un médecin |  |  |  |
| Un pharmacien | >>> Débloquer la section 6 |  |  |
| Un nutritionniste |  |  |  |
| Un physiothérapeute |  |  |  |
| Un naturopathe |  |  |  |
| Un herboriste |  |  |  |
| Un vendeur illégal |  |  |  |
| Autre* |  |  |  |

* Veuillez préciser : _______________________

1. Quels renseignements avez-vous demandés à cette ou ces personnes ressources ? Cochez toutes les réponses qui s’appliquent.

|  | Médecin | Nutritionniste | Conseiller SQDC | Conseiller d’une compagnie de cannabis | Physio-  thérapeuthe | Naturopathe | Herboriste |
| --- | --- | --- | --- | --- | --- | --- | --- |
| Un conseil sur la forme et la méthode de consommation du cannabis |  |  |  |  |  |  |  |
| Un conseil sur les variétés et les teneurs en THC/CBD disponibles sur le marché |  |  |  |  |  |  |  |
| Un conseil sur l’efficacité du cannabis pour soulager ou traiter une condition de santé |  |  |  |  |  |  |  |
| Un conseil sur les effets néfastes du cannabis |  |  |  |  |  |  |  |
| Un conseil sur la sécurité de combiner le cannabis avec mes médicaments |  |  |  |  |  |  |  |

1. Aimeriez-vous avoir accès plus facilement à des conseils de professionnels de la santé en lien avec votre usage de cannabis à des fins médicales?
   - Oui
   - Non
   - Je préfère ne pas répondre

Note : La section 6 n’est accessible que si le répondant a rapporté avoir consulté un pharmacien à la question 36.

Section 6 : Perception du rôle du pharmacien

1. Quel(s) renseignements avez-vous demandé(s) à votre pharmacien ? Sélectionnez toutes les réponses qui s’appliquent.

- Un conseil sur la forme et la méthode de consommation du cannabis.
- Un conseil sur les variétés et les teneurs en THC/CBD disponibles sur le marché.
- Un conseil sur l’efficacité du cannabis pour soulager ou traiter une condition de santé.
- Un conseil sur les effets néfastes du cannabis.
- Un conseil sur la sécurité de combiner le cannabis avec mes médicaments.

1. Étiez-vous satisfait des renseignements et des conseils obtenus auprès de votre pharmacien ?
   - Oui j’étais satisfait
   - Non je n’étais pas satisfait
   - Je ne sais pas
   - Je préfère ne pas répondre
2. Concernant votre satisfaction lors de votre entretien avec le pharmacien, sélectionnez tous les énoncés qui s’appliquent :
   - Le pharmacien a su répondre à toutes mes questions.
   - Le pharmacien n’avait pas les connaissances requises pour répondre à mes questions.
   - Le pharmacien m’a référer à une autre ressource professionnelle (ex : médecin, clinique de cannabis, compagnie fabricante) pour répondre à mes questions.
   - Les conseils du pharmacien ont dépassé mes attentes et mes questions.
   - Le pharmacien était ouvert à ma consommation de cannabis pour traiter ma condition de santé.
   - J’ai senti que le pharmacien a jugé négativement ma consommation de cannabis.
   - Le pharmacien a vérifié que le cannabis soit compatible avec mon profil de santé et avec mes médicaments.
   - Le pharmacien m’a encouragé à cesser ou à réduire ma consommation du cannabis pour traiter ma condition de santé.
   - Autre raison (veuillez préciser).

Conclusion : Nous vous remercions du temps que vous avez consacré à cette enquête. Votre participation nous apportera de précieux renseignements pour la réalisation de ce projet de recherche.

Au terme de ce questionnaire, si vous désirez discuter avec un intervenant à propos de votre consommation de cannabis, nous vous invitons à consulter votre médecin ou votre pharmacien. Les professionnels des CLSC et du Centre de réadaptation en dépendance de Québec peuvent aussi vous aider.

**Fin du questionnaire**

___________________________________________________________________________

Références :

1. Sexton M, Cuttler C, Finnell J, Mischley L. A Cross-Sectional Survey of Medical Cannabis Users: Patterns of Use and Perceived Efficacy. Cannabis and Cannabinoid Research. 2016;1(1):131-138.

2. Enquête québécoise sur le cannabis – 2019 [Internet]. Québec: Institut de la statistique Québec; 2019 [cited 13 August 2020]. Available from: https://www.stat.gouv.qc.ca/enquetes/sante/eqc-questionnaire-fr-2019.pdf

3. Centre de statistiques sur le cannabis [Internet]. Www150.statcan.gc.ca. 2019 [cited 13 August 2020]. Available from: https://www150.statcan.gc.ca/n1/pub/13-610-x/cannabis-fra.htm

4. Enquête canadienne sur le cannabis 2019 Rapport méthodologique [Internet]. Ottawa: Santé Canada; 2019 [cited 13 August 2020]. Available from: https://epe.lac-bac.gc.ca/100/200/301/pwgsc-tpsgc/por-ef/health/2019/130-18-f/rapport.pdf

5. Lucas P, Walsh Z. Medical cannabis access, use, and substitution for prescription opioids and other substances: A survey of authorized medical cannabis patients. International Journal of Drug Policy [Internet]. 2017;42:30-35. Available from: https://www-sciencedirect-com.acces.bibl.ulaval.ca/science/article/pii/S0955395917300130?fbclid=IwAR1E-25ufsfsJKfbMTCY2523qIRNrS0nZXxHIkj12-YZSTVUGOtFAMcOmFw

6. Free Online PROMIS-10 Score Calculator - OrthoToolKit [Internet]. Orthotoolkit.com. 2020 [cited 13 August 2020]. Available from: https://www.orthotoolkit.com/promis-10/
